# When pain persists after peroral endoscopic myotomy: endoscopic management of a large postprocedural intramural esophageal hematoma

**DOI:** 10.1055/a-2944-3460

**Published:** 2026-09-11

**Authors:** Mathieu Pioche, Alexandru Lupu, Florian Rostain, Jean Grimaldi, Roupen Djinbachian, Jérémie Jacques, Jérôme Rivory

**Affiliations:** 1 Gastroenterology and Endoscopy UnitEdouard Herriot Hospital, Hospices Civils de LyonLyonFrance; 2 Gastroenterology and Endoscopy Unit 25443 University Hospital of Montreal (CHUM)MontrealCanada; 3 Gastroenterology and Endoscopy Unit 37925 Centre Hospitalier Universitaire DupuytrenLimogesAquitaine-Limousin-Poitou-CharentesFrance

A 32-year-old man underwent posterior selective circular peroral endoscopic myotomy (POEM) for type II achalasia. The procedure was uneventful, without unusual bleeding, subcutaneous emphysema, or symptomatic pneumoperitoneum.

**Fig. 1:**
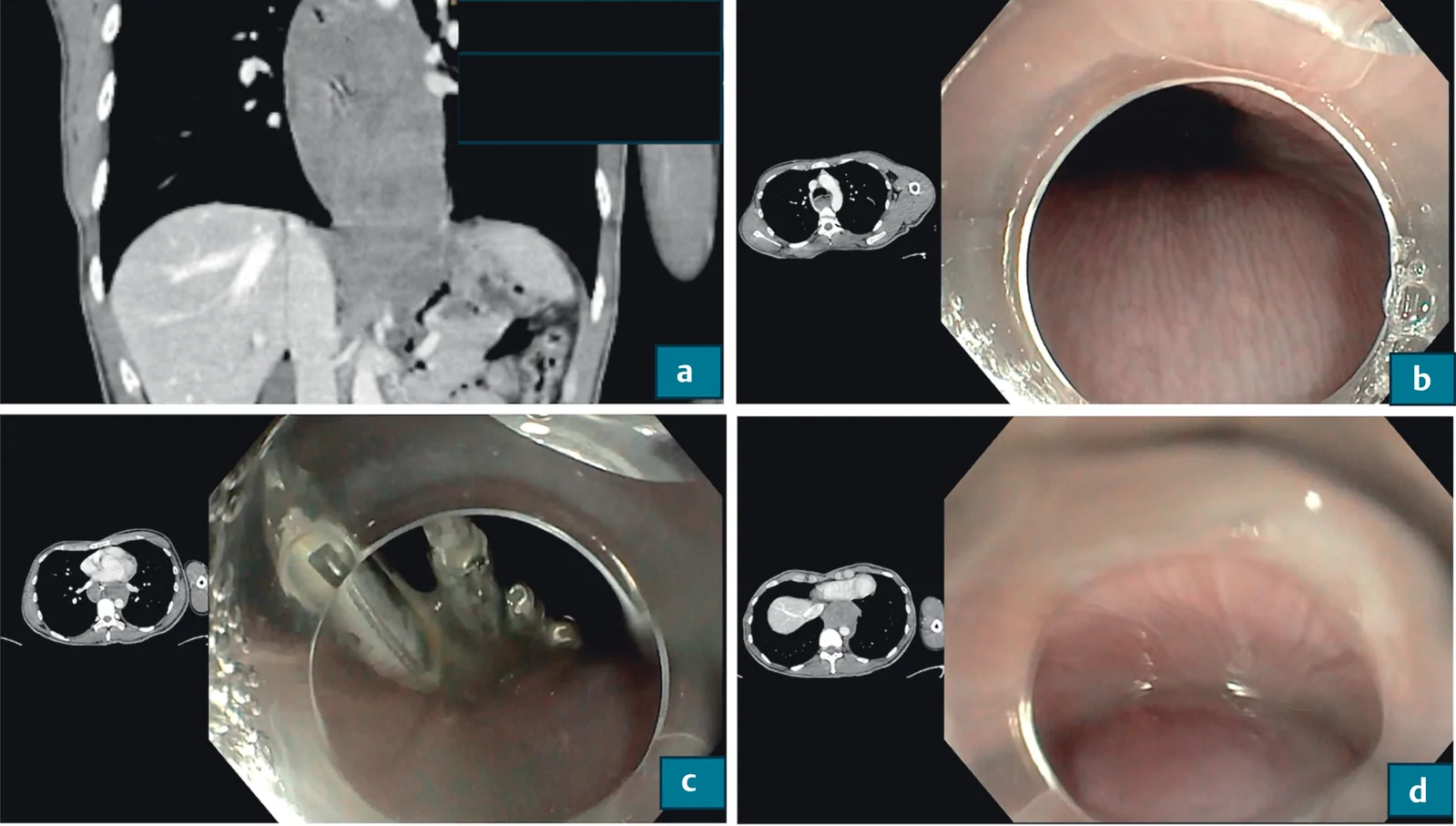
CT and endoscopic aspect of the hematoma: ( **a** ) a transversal CT scan aspect of the hematoma, ( **b** ) an endoscopic and CT view above the clips, ( **c** ) an endoscopic and CT view at the clip level; **1** : an endoscopic and CT view under the clips.

In the recovery room, the patient developed severe chest pain requiring nonsteroidal anti-inflammatory drugs. On postoperative day 1, persistent pain associated with a hemoglobin decrease of 1.5 g/dL, in the absence of overt gastrointestinal bleeding, prompted thoracoabdominal computed tomography (CT). CT revealed a large intramural esophageal hematoma occupying the entire submucosal tunnel and extending proximally within the submucosal layer, approximately 5 cm above the mucosal entry site ( Fig. 1–d ).

**Fig. 2:**
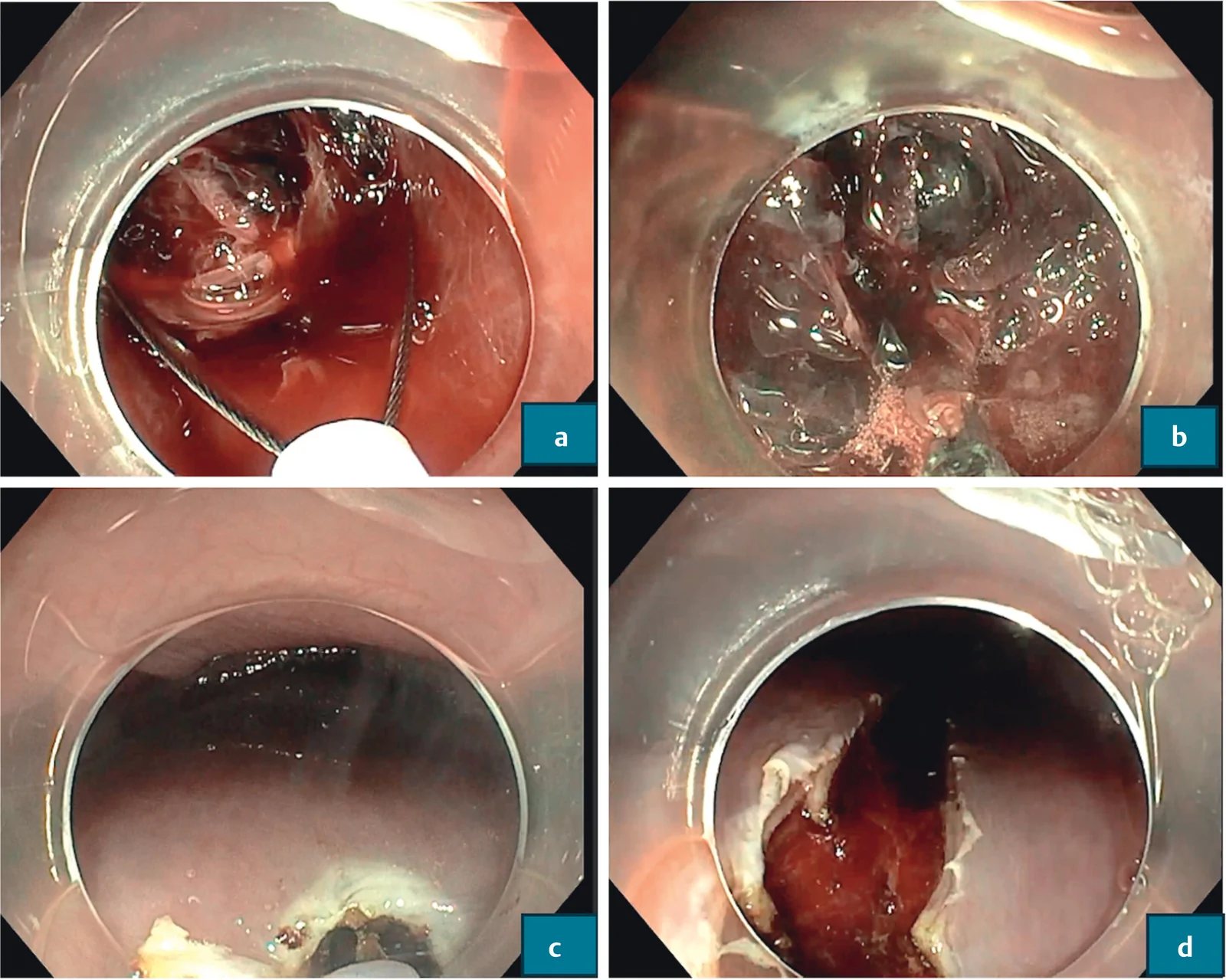
Endoscopic procedure for hemostasis and hematoma decompression. ( **a** ) The blood clot removal with snare, ( **b** ) hemostasis of the spurting vessel, ( **c** ) snare-tip incision of the submucosal hematoma, and ( **d** ) the final aspect.

Repeat endoscopy was performed immediately ( Video 1 , Fig. 2 ). The clips closing the mucosal entry were carefully removed to avoid tearing the tunnel opening. Large blood clots were extracted using a snare ( Fig. 2a ), exposing an actively spurting vessel that was successfully coagulated with hot biopsy forceps ( Fig. 2b ). Complete clot evacuation and extensive saline irrigation confirmed the absence of additional bleeding. The tunnel entry was then reclosed with clips.

**Video 1** When pain persists after peroral endoscopic myotomy: endoscopic management of a large postprocedural intramural esophageal hematoma.


Because the proximal submucosal dissemination of blood caused significant narrowing of the esophageal lumen, the hematoma was incised longitudinally using the snare tip ( Fig. 2c, d ) to facilitate drainage and potentially reduce the risk of secondary infection. 1​ A 5-day course of antibiotics was prescribed.

Pain markedly improved on the same day, oral feeding was resumed, and the patient was discharged the following day. No recurrence of symptoms or delayed infectious complications occurred during the follow-up.

Intramural esophageal hematoma is an uncommon complication after POEM 2​ 3​ ​4​ and has been reported only exceptionally in the literature. This case illustrates that persistent severe pain after POEM should prompt cross-sectional imaging and that early endoscopic evacuation of a large hematoma may provide rapid symptom relief and possibly prevent secondary infection.

Endoscopy_UCTN_Code_TTT_1AO_2AP
